# Role of abnormal energy metabolism in the progression of chronic kidney disease and drug intervention

**DOI:** 10.1080/0886022X.2022.2072743

**Published:** 2022-05-10

**Authors:** Xuyan Liu, Huasheng Du, Yan Sun, Leping Shao

**Affiliations:** Department of Nephrology, The Affiliated Qingdao Municipal Hospital of Qingdao University, Qingdao, China

**Keywords:** Energy metabolism, chronic kidney disease, hypoxia, mitochondrial dysfunction, drug intervention

## Abstract

Chronic kidney disease (CKD) is a severe clinical syndrome with significant socioeconomic impact worldwide. Orderly energy metabolism is essential for normal kidney function and energy metabolism disorders are increasingly recognized as an important player in CKD. Energy metabolism disorders are characterized by ATP deficits and reactive oxygen species increase. Oxygen and mitochondria are essential for ATP production, hypoxia and mitochondrial dysfunction both affect the energy production process. Renin-angiotensin and adenine signaling pathway also play important regulatory roles in energy metabolism. In addition, disturbance of energy metabolism is a key factor in the development of hereditary nephropathy such as autosomal dominant polycystic kidney disease. Currently, drugs with clinically clear renal function protection, such as Angiotensin II Type 1 receptor blockers and fenofibrate, have been proven to improve energy metabolism disorders. The sodium-glucose co-transporter inhibitors 2 that can mediate glucose metabolism disorders not only delay the progress of diabetic nephropathy, but also have significant protective effects in non-diabetic nephropathy. Hypoxia-inducible factor enhances ATP production to the kidney by improving renal oxygen supply and increasing glycolysis, and the mitochondria targeted peptides (SS-31) plays a protective role by stabilizing the mitochondrial inner membrane. Moreover, several drugs are being studied and are predicted to have potential renal protective properties. We propose that the regulation of energy metabolism represents a promising strategy to delay the progression of CKD.

## 1. Introduction

Chronic kidney disease (CKD) has been identified as a leading public health problem worldwide, with an estimated global prevalence of CKD is 13.4% (11.7–15.1%) [1]. CKD refers to the syndrome that is characterized by the decreased glomerular filtration rate (GFR) over three months caused by various etiologies, accompanied by metabolic disorders and clinical symptoms. End-stage kidney disease (ESKD) can be diagnosed when the GFR is below 15 mL/min per 1.73 m^2^, which has a severe prognosis and is one of the major life-threatening diseases. The major causes of CKD include glomerulonephritis, diabetic kidney disease (DKD), hypertension nephropathy, and cystic kidney disease, etc. [2], and approximately 45% of all cases of ESKD are caused by DKD [3]. But the development and progression of the CKD remain to be fully elucidated.

Energy metabolism is one of the most fundamental functions of life. Therefore, the normal and orderly energy metabolism is the biochemical basis for the maintenance of the specific structure and physiological function of the kidney. It can result in dysfunction of kidney, and eventually CKD, if energy metabolism is disordered or altered for a variety of reasons, like mitochondrial dysfunction brought by diabetic nephropathy.

In this review, we overview the functions of renal energy metabolism and discuss the role of energy metabolism disorders in the development and progression of CKD. In addition, we highlight potential therapeutic strategies for energy metabolism disorders that could be beneficial to CKD.

## 2. Renal energy metabolism under physiological conditions

The kidney is an organ rich in mitochondria and high energy metabolism. The majority of ATP is produced through oxidative phosphorylation (OXPHOS) in healthy kidney [4], and the fuels for OXPHOS are multitudinous, including fatty acids, glucose, lactic acid, amino acids, etc. The substance preferences at different renal sites reflect the demand for ATP at these areas, for instance, the glomeruli tend to use glucose, while the renal tubule tends to use fatty acid. In general, fatty acid oxidation is the preferred energy source for hypermetabolic tissues, because in the case of sufficient oxygen, oxidation of fatty acid, such as palmitic acid, produces 106 molecules of ATP per fuel molecule, even more efficient than glucose oxidation *via* OXPHOS (produces 36 molecules of ATP per glucose molecule) or glycolysis (produces 2 molecules of ATP per glucose molecule) [5]. Proximal tubules not only use fatty acids to generate energy, but also store energy in the form of gluconeogenesis [6]. Furthermore, the majority of ATP produced by kidney is used by epithelial cells to power tasks, for example, solute transport facilitated by Na^+^-K^+^-ATPase activity coupled with high-affinity transporters consumes at least 50% of renal ATP production under physiological conditions [7,8].

Renal oxygenation is defined as the relationship between renal oxygen delivery (DO2) and renal oxygen consumption (QO2). High blood flow to the kidneys enjoys approaches 20% of cardiac output [9,10], which is three times higher than myocardial blood flow [11]. The high renal blood flow (RBF) can maintain an adequate DO2 level for normal energy metabolism. The kidney has a high QO2 per gram of tissue, second only to the heart (2.7 *vs.* 4.3 mmol/kg/min for the heart) [12]. It is known from numerous experimental studies that the filtered solute load is the major determinant of renal QO2 [13]. Increased RBF can elevate DO2, as well as GFR and the filtered solute load that will then increase renal QO2, since the reductions of GFR and the filtered solute load act to decrease tubular solute reabsorption and renal QO2 [11,14]. In addition, the corticomedullary junction and the outer medulla of kidney remain in a state of physiologically hypoxic despite the large amount of blood the organ receives [15], which is primarily the result of an arterial-to-venous diffusional oxygen shunt [16–19]. Therefore, characteristic of renal oxygen supply predisposes the kidney to hypoxic damage.

**Figure 1.**
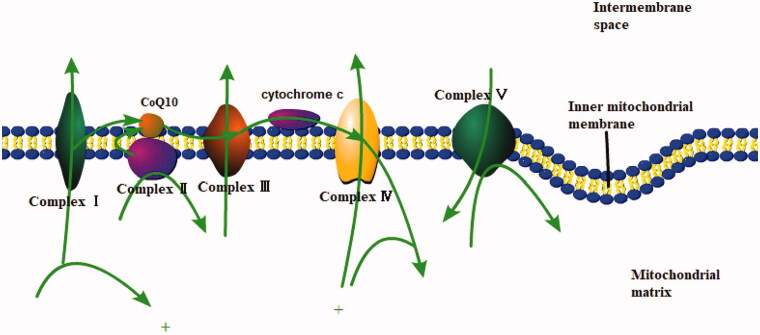
Mitochondrial electron transport chain.

Mitochondria are involved in many cellular processes and are present in all cells except mature red blood cells. The most important function of mitochondria is to produce ATP *via* OXPHOS, which plays an important role in the kidney with high energy requirements. Mitochondria are also engaged in metabolic pathways, such as the β-oxidation and the urea cycle. Moreover, they play a significant role in heat production, calcium homeostasis, and the control of intrinsic apoptotic pathways. The key to produce ATP is the electron transport chain located in the inner membrane of the mitochondria, which consists of complex I, II, III, IV, coenzyme Q and cytochrome C (Figure 1). Complex I, II, III and IV transfer electrons from high-energy compounds, NADH and FADH2, to molecular oxygen. The electrochemical gradient generated in the process is used by ATP synthase (also known as complex V) to synthesize ATP, in the meantime approximately 1–2% of the oxygen consumed receive electrons directly from complexes I and III to form reactive oxygen species (ROS) [20]. Mitochondrial DNA (mtDNA) refers to the mitochondria’s own DNA, which does not follow the classic Mendelian rules of inheritance and is often inherited from the mother. Except that complex II contains only nuclear DNA (nDNA)-encoded subunits, the respiratory chain complex consists of mtDNA-encoded subunits and nDNA-encoded subunits [21]. The electron carrier coenzyme Q10 (CoQ10) is a small lipophilic molecule, which shuttles electrons from complexes I and II to complex III [22]. And CoQ10 is also a key antioxidant and a modulator of the mitochondrial permeability transition pore [22]. The main function of cytochrome c is the transmission of electrons from complex III to complex IV of the respiratory chain [23].

As previously mentioned, ATP produced by the kidneys is mainly used for reabsorption, which is primarily accomplished by renal cortical cells, including proximal tubule cells (PTCs) and the cells of the thick ascending loop of Henle (TAL). Studies have shown that there is a significant decrease in oxygen concentration between the renal cortex and medulla [24]. In addition, animal experiments showed that PTCs and TAL cells contain most renal mitochondria and consume most oxygen in the kidney [6]. In recent years, podocytes that form the glomerular filtration barrier have also been known to have high energy requirements and abundant mitochondria [25].

## 3. Energy metabolism disorders contribute to chronic renal failure

### 3.1. Extracellular ATP

**Figure 2.**
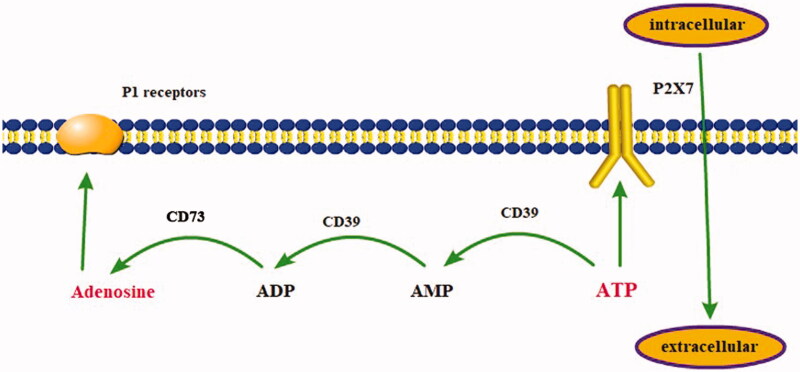
Extracellular purinergic catabolic and signaling pathways. *Abbreviations*: ecto-nucleoside triphosphate diphosphohydrolase (CD39); ecto-5′-nucleotidase (CD73); AMP: adenosine monophosphate; ADP: adenosine diphosphate.

ATP is the most vital source of cellular energy for biologic systems, and it exists at high concentrations (3–5 mM) within the cells and micromolar concentrations outside the cells. The release of extracellular ATP (eATP) can be triggered by a wide range of stimuli such as mechanical stress, cell membrane damage, inflammation, hypoxia, and excitation of neural tissue, and cell growth and death [26]. Under physiological conditions, extracellular ATP is successively hydrolyzed by the ecto-nucleoside triphosphate diphosphohydrolase into adenosine diphosphate (ADP) and adenosine monophosphate, which is then hydrolyzed by ecto-5′-nucleotidase (CD73) to adenosine (Figure 2) [27]. Growing evidence suggests that eATP signaling and adenosine signals may be involved in a variety of diseases leading to chronic renal failure, including DKD [28,29], hypertensive nephropathy [30], polycystic kidney disease (PKD) [31,32] and so on.

Release of ATP from the cell allows it to initiate various extracellular purinergic signaling pathways. Extracellular ATP and ADP interact with the purinergic P2 receptors to promote inflammation. P2 receptors are subdivided into to two subclasses: the G protein-coupled P2Y receptors and the ATP-gated P2X nonselective ion channels. ATP primarily activates NLRP3 inflammasome through binding P2X7 receptors (P2X7R), resulting in secretion of IL-1β. In addition, ADP may stimulate IL-1 β production through the P2Y receptor expressed on macrophages [33]. In individuals with DKD, P2X7R and NLPR3 were upregulated compared with controls [26], and the increased expression of NLRP3 and IL-18 release were attenuated by P2X7R silencing in murine podocytes in another study [34]. The role of P2 receptors in renal fibrosis has been investigated in the unilateral ureteral obstruction model, which showed that TGF-β expression, macrophage infiltration and renal tubular fibrosis were reduced in P2X7R knockout mice compared with wild-type mice.

Adenosine signals through four G protein-coupled P1 receptors termed A_1_ receptor (A_1_R), A_2A_ receptor (A_2A_R), A_2B_ receptor (A_2B_R), and A_3_ receptor (A_3_R). Among them, A_1_R and A_2A_R have high affinity for their ligands, while A_2B_R and A_3_R require increased adenosine levels to mediate cellular responses [29]. Functionally, A_2A_R attenuates inflammation and improves the protective effect of renal fibrosis in the CKD model [27], and absence of A_1_R results in diabetic ultrafiltration and increased glomerular damage, suggesting that A_1_R also plays a protective role [29]. In contrast, chronic A_2B_R signaling promotes renal fibrosis. CD73 expression and adenosine were elevated in diabetic mice [35], and renal biopsy samples from patients with CKD showed elevated levels of CD73 and A_2B_R mRNA [30]. Fibroblasts, key cells in the fibrosis process, express CD73 and A_2B_R, whose chemical activation contributes to increased transcription of pro-fibrosis and inflammatory mediators, including α-actin 2, IL-6, and TGF-β [36]. Adenosine and eATP signaling pathways mainly activate downstream inflammatory and renal fibrosis signals by binding to P2 and P1 purine receptors, and play a significant role in the development of CKD.

### 3.2. Renal hypoxia

Oxygen is one of the essential substances in OXPHOS, and hypoxia contributes to energy metabolism disorders in cells. It has been validated by numerous studies that chronic hypoxia of the renal tubules has been postulated as a final common pathway in the development of ESKD [37–39]. Generally speaking, anoxia has two kinds of mechanisms: one is the relative hypoxia caused by increased oxygen consumption, and the other is the absolute hypoxia due to insufficient oxygen supply.

Renal hyperfiltration is an early characteristic of DKD [40]. Hyperglycemia causes afferent arteriolar dilatation by releasing vasoactive mediators [41], increasing RBF, and thereby increasing salt and fluid filtration by the kidneys. Ultrafiltration increases the transport load and energy consumption of renal tubules, which increases the consumption of oxygen [42]. Studies have demonstrated that a significant decrease in oxygen tension was detected in the renal cortex in a type 1 diabetes mellitus rat model [43]. Increased oxidative stress is also responsible for the increased oxygen consumption [2]. Mitochondrial dysfunction caused by various reasons is an important reason for the increase of ROS [44]. Excessive ROS will be neutralized by reducing substances in the body, such as nitric oxide (NO), which is also a suppressor of mitochondrial respiration. The depletion of NO may stimulate mitochondrial respiration and increase oxygen consumption, resulting in tissue hypoxia [45].

A variety of chronic diseases cause an imbalance of vasoactive substances, resulting in glomerular injury and vasoconstriction of efferent arterioles, reducing peritubular capillary blood flow, leading to renal tissue hypoxia and progressive loss of renal function [39]. Among a variety of vasoactive substances, local activation of renin-angiotensin system (RAS) is particularly important, which will be described in detail later. When kidney function declines to a certain extent, its production of erythropoietin (EPO) decreases, resulting in renal anemia, further aggravating the symptoms of hypoxia. Furthermore, in acute kidney injury (AKI) caused by ischemia-reperfusion, inadequate oxygen supply for kidney is the root of all cell damage.

Researches show that glucose oxidation increases in the proximal tubule when oxygen delivery is compromised in the diabetic environment, but the proximal tubule prefers fatty acid oxidation under physiological conditions [6,46]. Hypoxia also induces upregulation of hypoxia-inducible factor (HIF), which mediates the transition from oxidative metabolism to glycolysis, which can enhance cell survival by providing ATP in an oxygen-independent manner [47]. Tracer studies in rats have shown that reducing oxygen supply increases lactate and alanine formation from pyruvate, suggesting that energy metabolism of the kidney will be transformed from OXPHOS to glycolysis in hypoxic environment [48].

Notably, renal PTCs have very limited capacity of ATP production *via* anaerobic glycolysis because they do not have high concentration of glycolytic rate-limiting enzymes [6]. When hypoxia exceeds its regulatory capacity, renal tubule cells can be transformed into myofibroblasts by epithelial-mesenchymal transdifferentiation [49]. In addition, mitochondrial dysfunction occurs in cells are exposed to severe or prolonged hypoxia, releasing a large number of ROS, leading to kidney inflammation and fibrosis, and exacerbating cell apoptosis and necrosis [50,51]. In conclusion, hypoxia leads to tubule fibrosis and loss of peritubular capillaries, which in turn exacerbates hypoxia, setting in train a vicious cycle.

### 3.3. RAS

RAS is an important humoral regulation system in the body, whose components including renin, angiotensinogen, angiotensin converting enzyme and angiotensin II (Ang II). Ang II is considered the major physiologically active component of RAS, and it can directly constrict vascular smooth muscle cells and stimulate aldosterone production, which is mediated through Ang II type 1 receptor activation and contributes to the development of hypertension [52]. Local activation of RAS is especially significant for the kidney, because it can lead to efferent arterioles constriction, peritubular capillaries hypoperfusion, and subsequent tubulointerstitium hypoxia [39]. Animal experiments revealed that the mice injected with Ang II were found having the microvascular injury with peritubular capillary loss and focal loss of endothelial nitric oxide synthase (eNOS) [53]. NO that is synthesized by eNOS is an endothelium-dependent vascular relaxant, and the absence of NO can exacerbate Ang II-induced vasoconstriction. Moreover, NO is also a mitochondrial respiratory suppressor, and its reduction can result in inefficient cellular respiration and hypoxia [45]. Angiotensin II can also up-regulate CD73 expression, increase adenosine content in kidney, and activate A_2B_R on renal mesangial cells and fibroblasts to promote fibrosis [33]. Ang II induces the increase expression of nicotinamide adenine dinucleotide phosphate oxidase, producing superoxide anions that result in mitochondrial uncoupling, i.e. increased oxygen consumption for each ATP produced, which not only aggravates kidney hypoxia, but also increases ROS production [54]. Studies have shown that the angiotensinogen gene is stimulated by NF-κB activation, which is sensitive to the redox ratio, providing a positive feedback loop that can upregulate angiotensin II production [55]. Therefore, RAS induces energy metabolism disorders *via* both hemodynamic and nonhemodynamic mechanisms, resulting in CKD.

### 3.4. Mitochondrial dysfunction

The characteristic of mitochondrial dysfunction is reduced ATP generation and increased ROS production, which is present in various kidney diseases. Mitochondrial dysfunction includes inherited and acquired, inherited mitochondrial dysfunction such as nuclear gene or mtDNA mutations is more serious. Most of these hereditary mitochondrial diseases affect the whole system, and more than 50% of patients suffer from renal insufficiency [56]. Autosomal dominant polycystic kidney disease (ADPKD) is one of the most common monogenetic diseases [57] and is characterized by the development of large fluid-filled renal cysts, with approximately 50% of ADPKD patients progressing to ESKD [58]. The most common cause of ADPKD is mutations in two genes, PKD1 and PKD2, which encode polycystin 1 and polycystin 2, respectively. Polycystins affect the function and morphology of mitochondria [59,60], and the loss of polycystin 1 alters the calcium transport in mitochondria. Calcium dyshomeostasis results in increased mitochondrial ROS production, which cause biomolecular modifications of DNA (nDNA and mtDNA) and proteins, leading to lipid peroxidation, and ultimately to the opening of mitochondrial permeability transformation pores and the loss of mitochondrial membrane potential [61]. Fatty acid β oxidation was reduced in renal tubule-specific PKD1 mutated mice [62], and transcription of hexokinase 2, one of the key enzymes of glycolysis, is upregulated in PKD1-deficient cells [63]. Of PKD1-null tubule epithelial cells, mitochondrial metabolism genes were significantly down-regulated, while nucleic acid and protein metabolism genes were up-regulated [64]. It has been demonstrated from different aspects that mitochondrial dysfunction leads to changes in the original energy metabolism mode, and glycolysis and other metabolic modes that do not require mitochondrial participation are significantly increased. Another hereditary mitochondrial dysfunction, mutations in mitochondrial carnitine O-palmityl transferase 2, may also lead to CKD. Mutations in carnitine O-palmityl transferase 2, an enzyme that transport fatty acids to mitochondria, lead to abnormal function in cells that rely on fatty acid oxidation for energy, such as renal tubules, ultimately leading to the progression of kidney disease [65]. Besides, defects in CoQ10 biosynthesis are a major cause of focal segmental glomerulosclerosis in children [21]. Lack of CoQ10 reduces respiratory chain activity and impairs cell energy generation, and different gene mutants involved in coenzyme Q10 biosynthesis will lead to different results: COQ2 mutant has defect in ATP synthesis and significantly increased ROS production, while PDSS2 mutant only show significant reduction in ATP synthesis [66]. When mitochondria are dysfunctional, the energy metabolism pathway that generates ATP through mitochondria is blocked, and the glycolysis pathway is increased. At the same time, mitochondrial dysfunction also leads to increased ROS production, which eventually results in CKD.

Acquired mitochondrial dysfunction refers to the non-genetic diseases usually caused by hazardous environmental factors, such as high blood sugar and acute ischemia. Mitochondrial dysfunction in the kidney is mainly manifested as the dysfunction of proximal tubule, TAL and podocyte according to the distribution characteristics of mitochondria. The generation of ROS by damaged or dysfunctional mitochondria has been postulated as the primary initiating event in the development of diabetes complications [67]. Chronic hyperglycemia causes structural alterations of proteins through the Maillard reaction. As mentioned earlier, hyperglycemia can contribute to hypoxia in the kidneys by increasing ultrafiltration, which leads to an increase in glycolysis. Methylglyoxal, the product of glycolysis, has also turned out to be elevated in diabetics. Post-translational modification of mitochondrial proteins by methylglyoxal is speculated to be the cause of decreased activity of respiratory complex III and increased production of ROS in DKD [68]. Under the physiological state, glomerular cells depend on glucose oxidation and renal tubular cells depend on fatty acid oxidation for energy production, but these are disrupted in diabetes. These cells are forced to use alternative fuels, such as fatty acid oxidation in podocytes and glucose oxidation glycolysis in PTCs, resulting in lipid toxicity and glycotoxicity, which are presumed to damage mitochondria in the cytoplasm and increase ROS production. Another common disease that causes CKD is AKI [69], whose familiar clinical etiology is septic, ischemic, and toxic. Ischemic and toxic forms of AKI are characterized by prominent mitochondrial fragmentation [21], and the proximal tubule is a primary site for mitochondrial disruption [70]. ROS released from damaged mitochondria contribute to the oxidative stress in AKI [21].

Elevated ROS can partially activate another protective mechanism, autophagy, which is an intracellular degradation system for cellular homeostasis [25]. Nonselective autophagy can be used for amino acid recovery, protein synthesis, and ATP production during nutrient deficiencies. Selective autophagy, such as mitophagy (remove damaged mitochondria), lysosomal autophagy (remove damaged lysosomes), can occur to remove intracellular toxic substances in certain stressed situations. Growing evidences support the viewpoint that disturbance of autophagy/mitophagy is associated with the pathogenesis of renal diseases such as AKI, DKD and glomerulosclerosis [71–73]. Autophagy disorders further aggravate ROS production, and excessive ROS can destroy a variety of functional enzymes, mitochondrial proteins and mtDNA, resulting in the change of ATP synthesis and inducing mitochondrial membrane permeability transition [74]. Electron microscopy studies have shown that mitochondria swelling was found in the proximal tubules of humans and mice with DKD [75,76].

Mitochondrial damage not only impairs energy production, but also increases ROS production, which further aggravates mitochondrial damage. Persistent oxidative stress is known to play a significant role in the progression of many diseases to CKD [6,77]. ROS can activate NLRP3 inflammasome and up-regulate the expression of cytokines IL-18, IL-1β, TGF-β, and NF-κB, resulting in renal pyroptosis and renal fibrosis[78,79]. In addition, cytochrome C released by fragmented mitochondria also induces cell apoptosis [80].

These pathways of energy metabolism disorders do not exist in isolation. Generally, they co-exist and interact in the body. The ultimate result is renal inflammation and pyroptosis, glomerulosclerosis and tubular fibrosis, finally leading to CKD. In the following, we will present the current status of drug intervention for energy metabolism disorders according to the above-mentioned pathways.

## 4. The drug intervention of energy metabolism disorders

### 4.1. SGLT2 inhibitors

The sodium-glucose co-transporter 1 and sodium-glucose co-transporter 2 (SGLT2) belong to the SLC5 active glucose transporters family, and their main role is to mediate glucose reabsorption in the kidney. SGLT2 is mainly located in the S1 segment of the proximal convoluted tubule and is responsible for 90% of renal glucose reabsorption [81,82], while sodium-glucose co-transporter 1 is located in the S2/S3 segment of the proximal convoluted tubule and reabsorbs about 10% of the filtered glucose [83]. Glomerular hyperfiltration and reduced renal tissue PO2 occur in the early stages of type 1 diabetes mellitus or type 2 diabetes mellitus (T2DM), which is a risk factor for DKD progression to ESKD [43,84]. Multiple studies in diabetic rodent models [85] and in patients with T2DM [86] have reported hyperglycemia increases the expression of SGLT2 in the kidney, resulting in increased glucose reabsorption. Studies have shown that angiotensin II type 1 (AT1) receptor has been implicated in the upregulation of SGLT2 expression in diabetes [87]. Hyperreabsorption of glucose contributes to increased energy-consuming transport of proximal renal tubule cells, accompanied by a significant increase in renal oxygen demand, ultimately inducing hypoxia and cellular stress [88]. Increased reabsorption by co-transport of sodium reduces sodium availability at the macula densa, inactivates tubule-glomerular feedback, and induces vasodilation of afferent arterioles, increasing glomerular pressure, and promoting glomerular hyperfiltration. Concomitantly, increased renin secretion promotes vasoconstriction of efferent arterioles, further aggravating the intraglomerular pressure and hyperfiltration. Ultimately, compensatory mechanisms lead to glomerular hypertrophy [88].

Accumulated evidence in the past few years clearly suggests that SGLT2 inhibitors not only have a good hypoglycemic effect, but also have a strong renal protection effect. Data from the T2DM clinical trial suggest that SGLT2 inhibitors reduce albuminuria or proteinuria by 30-50%, with a significant benefit on ESKD [89–91]. Representative drugs for SGLT2 inhibitors are dapagliflozin, canagliflozin, and empagliflozin, all of which are approved in the United States and the European Union. The main mechanism of SGLT2 inhibitors as nephroprotective drugs is effectively reduce glucose reabsorption with the increased sodium concentration in the macula densa. This reinstates the tubule-glomerular feedback that supports afferent arteriolar contraction, and is associated with reduced renal energy demand and oxygen consumption, as well as a decrease of intraglomerular pressure and GFR [43,91,92]. Moreover, long-term reduction of hyperglycemia has been shown to positively affect oxidative stress levels, improve inflammatory processes, and slow the progression of renal tubule fibrosis [93]. It has been proposed that SGLT inhibitors can promote the restoration of myofibroblasts to erythropoietin producing fibroblasts by reducing oxidative stress, thereby increasing serum erythropoietin and hemoglobin, and further enhancing renal oxygen delivery [94]. Another mechanism of increased erythrocyte has been suggested by a study in T2DM, treatment with Dapagliflozin resulted in a reduction of hepcidin, a recognized inhibitor of erythropoiesis, which has been found increased in inflammatory conditions [95]. It should be noted that SGLT2 inhibitors have also been reported to improve hypoxia in the proximal tubule, but increase solute delivery in the distal nephron segments, and enhance medullary tubular transport burden and oxygen consumption, leading to acute tubule injury [96].

Interestingly, a recent study has been reported that SGLT2 inhibitors have been shown to alleviate chronic renal insufficiency induced by adenine in rats [97]. This demonstrates that the renal protective effect of SGLT2 inhibitors is not limited to diabetic nephropathy, but also non-diabetic nephropathy.

### 4.2. ARBs

Clinical practice has shown that angiotensin II type 1 receptor blockers (ARBs) have obvious renal protection and can delay the development of CKD [98–100]. Numerous experiments demonstrated that although ARBs could not reduce all-cause mortality, they could reduce the risk of ESKD [101,102]. The main renoprotective mechanisms of the ARBs are the inhibition of renal vasoconstriction and oxidative stress caused by Ang II, thereby correction of chronic hypoxia [103]. Under normal conditions treatment with an ARBs dilates both afferent and efferent arterioles at the same time [52]. However, patients with renal lesions generally have a compensatory effect of the body, which is to increase the intraglomerular pressure and GFR by dilating the afferent arterioles. Therefore, ARBs mainly dilate the efferent arterioles of the kidney [52,104]. ARBs-induced renal vasodilation results in an increase in RBF, improving renal ischemia and hypoxia. Yuko Izuhara treated mice with olmesartan (ARBs) or nifedipine (Calcium antagonist) or atenolol (Beta blockers) for 20 weeks, and despite similar BP reduction, only olmesartan significantly reduced proteinuria and prevented glomerular and tubulointerstitium damage (mesangial cell activation, podocyte injury, tubulointerstitial injury and inflammatory cell infiltration) [103]. Further experiments confirm unique renoprotective properties of ARBs, independent of reduction of blood pressure by dilating blood vessels but related to the reduction of oxidative stress (hydroxyl radicals scavenging and inhibition of the Fenton reaction) [103]. Notably, blocking the AT1 pathway reduces proximal fluid reabsorption and alters the expression of key sodium transporters in mice with AT1 receptor deficiency in the renal tubules, providing substantial protection to the kidney. The combination of RAS blocking and inhibiting SGLT2 had additional renal protective effects compared with either drug alone [105,106].

### 4.3. CoQ10

CoQ10 deficiency can be primary defects caused by mutations in genes involved in CoQ10 biosynthesis, or secondary defects caused by mutations in genes unrelated to CoQ10 biosynthesis [107]. Secondary CoQ10 deficiency, which has been described in patients with mitochondrial myopathy [108], mitochondrial DNA deletion syndrome [109], etc, has a higher incidence than primary CoQ10 deficiency [107]. The current clinical practice can prove that CoQ10 defect is the only OXPHOS disorder that can be clinically improved after oral CoQ10 supplementation with limitation of neurological and renal manifestations, amelioration of muscular symptoms and attenuation of histological alterations [110]. However, it is important to note that treatment can prevent the development of clinical manifestations, but once severe renal damage is established, it cannot be recovered [111]. *In vitro* experiments, the mechanism of CoQ10 is that exogenous CoQ10 reaches the mitochondrial intima, where it reactivates the electron flow within the mitochondrial respiratory chain [112,113]. In patients diagnosed with CoQ10 deficiency, renal function gradually returned to normal after oral supplementation with CoQ10, urinary protein excretion decreased, and albumin increased until normal [111,114].

### 4.4. SS-31

Mitochondrial dysfunction plays an important role in the occurrence and development of CKD. SS-31, also known as MTP-131 or elamipretide, is a tetrapeptide that accumulates in the inner membrane of mitochondria, which reduces ROS and prevents mitochondrial dysfunction [115]. SS-31 binds and stabilizes cardiolipin in the inner mitochondrial membrane and inhibit cardiolipin peroxidation [116]. Cardiolipin is a mitochondria-specific phospholipin, which is required for proper cristae formation [117], and serves as the scaffold for assembling respiratory complexes to facilitate electron transfer [118–121]. It also anchors cytochrome C to the inner mitochondrial membrane [122,123], and as we mentioned above, cytochrome C can also promote electron transfer from complex III to complex IV under physiological conditions. However, when the concentration of ATP decreases under pathological conditions such as ischemia, cardiolipin binds tightly to cytochrome C, resulting in damage to the spatial structure of cytochrome C and conversion from electron carrier to peroxidase, which catalyzes the oxidation of cardiolipin [124–126]. Studies have found that both DKD and AKI have cardiolipin peroxidation, which eventually results in mitochondrial dysfunction and reduced ATP synthesis capability [116,127]. In addition, these studies also found that changes in mitochondrial membrane permeability can lead to mitochondrial swelling, which releases cytochrome C into the cytosol [127]. This affects the electron transport of the mitochondrial respiratory chain and causes cell apoptosis [80].

SS-31 can selectively target the mitochondrial inner membrane and combine with cardiolipin to form a complex, which plays a mitochondrial protective role by inhibiting the activity of cytochrome C peroxidase [116,127] while increasing electron flux and reducing electron leakage in the respiratory chain, which then promotes mitochondrial ATP production [128].

### 4.5. HIF

As mentioned above, hypoxia is considered to be a common pathway in the development of ESKD. However, in the initial stage of hypoxia, the body performs a series of changes in response to the challenge of hypoxia, the most important of which is the upregulation of HIF.HIF is a protein heterodimer that is composed of one of the alternative oxygen-regulated α subunits (HIF-1α, HIF-2α, or HIF-3α) and a constitutively expressed subunit HIF-β [47]. Different α subunits enable HIF to have different roles. HIF-1 not only mediates the transition of oxidative metabolism to glycolysis [47], reducing oxygen consumption, but also mediates the subunit conversion of cytochrome C oxidase under hypoxia conditions, improving electron transport efficiency [129]. HIF-2 is a major regulator of EPO production [130] and vessel remodeling in diseases [131,132]. Anemia resulting from inadequate EPO production is a common complication of CKD [133]. In contrast, the function of HIF-3 remains largely unknown and controversial. In the kidney, HIF-1 is mainly expressed in renal epithelial cells, while HIF-2 is mainly expressed in renal interstitial fibroblast-like cells and endothelial cells.

**Figure 3.**
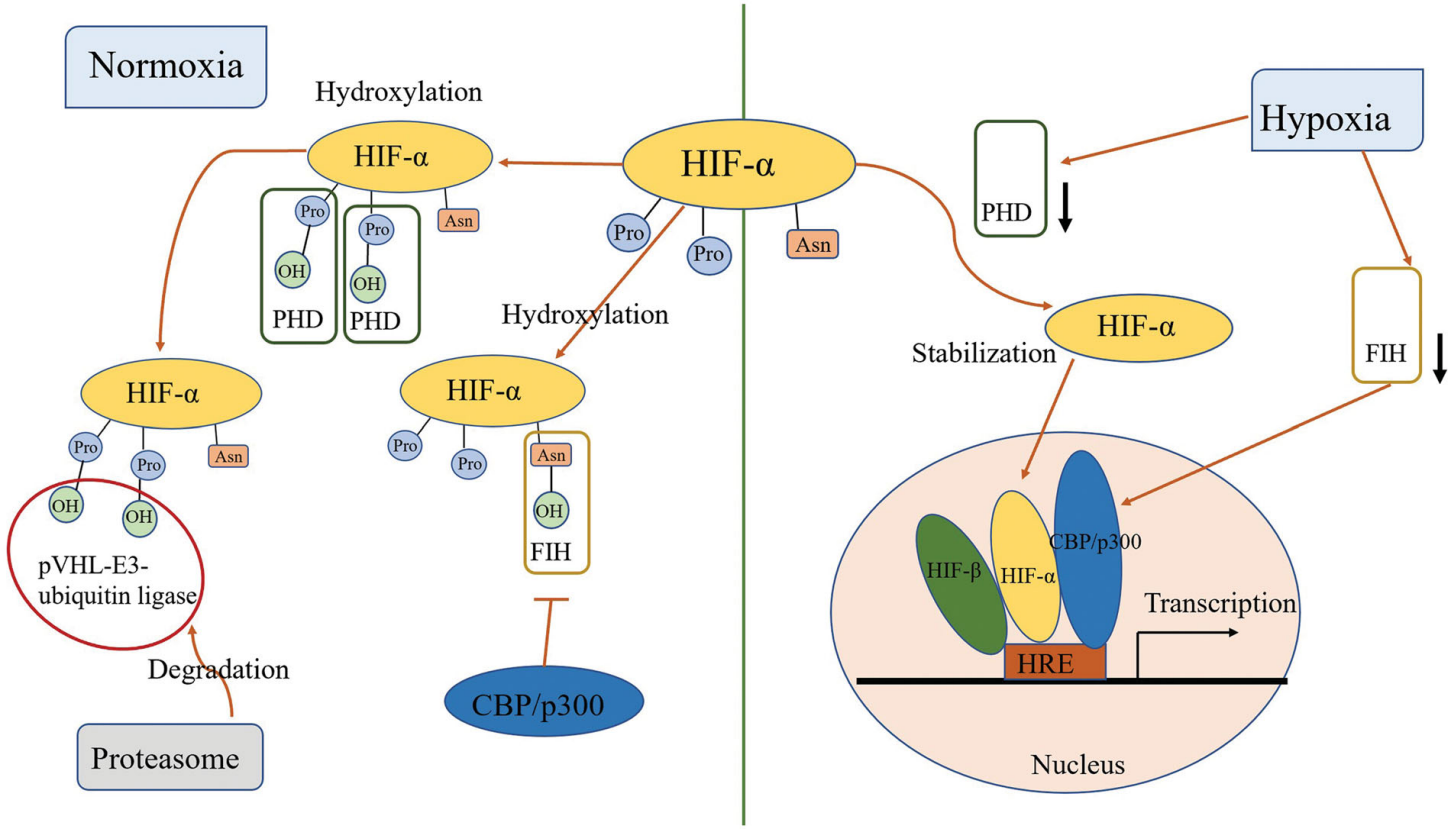
Regulation of the stability and transcription activity of HIF. Under normoxic conditions, two key proline residues (Pro) of HIF-α are hydroxylated by specific PHD. Following prolyl hydroxylation, HIF-α binds to the pVHL-E3-ubiquitin ligase complex and is rapidly degraded by the proteasome. Meanwhile, FIH hydroxylates the asparaginyl residue (Asn) of HIF-α to prevent the recruitment of CBP/p300 coactivator. During hypoxia, HIF-α becomes stable and moves from the cytoplasm to the nucleus, where it forms a HIF complex with HIF-β and binds to target gene to activate its transcription. Abbreviations: hypoxia-inducible factor (HIF), prolyl hydroxylase domain-containing protein (PHD), von Hippel-Lindau protein (pVHL), factor inhibiting HIF (FIH), CREB-binding protein (CBP), hypoxia response element (HRE).

HIF-α degrades rapidly under normoxic conditions, making it essentially undetectable. During hypoxia, HIF-α becomes stable and moves from the cytoplasm to the nucleus, where it forms a HIF complex with HIF-β and binds to the target gene to activate its transcription (Figure 3) [134,135]. The stability of HIF is mainly controlled by its post-translational modifications, such as hydroxylation, ubiquitination, and phosphorylation [136]. Under normoxic conditions, two key proline residues of HIF-α are hydroxylated by specific proline hydroxylase domain protein (PHD). Following prolyl hydroxylation, HIF-α binds to the von Hippel-Lindau protein-E3-ubiquitin ligase complex and is rapidly degraded by the proteasome [137,138]. PHDs are also divided into three subtypes, namely PHD1, PHD2, and PHD3. And PHD2 is a hydroxylase necessary for HIFα degradation under normal oxygen conditions [139,140]. In addition to PHDs, factor inhibiting HIF (FIH) hydroxylates the asparaginyl residue of HIF-α in the presence of oxygen to prevent the recruitment of CREB-binding protein/p300 coactivator, which is required for HIF to have full transcriptional activity [141,142]. Thus, PHDs control the stability of HIFα, and FIH regulates HIF transcriptional activity.

Pretreatment with PHDS inhibitors activated HIF and upregulated the expression of target genes, significantly reducing renal ischemic loss [143,144]. Although HIF can be activated in CKD, it is not sufficient because HIF is inhibited by oxidative stress and uremia [145]. PHDS inhibitors can help activate HIF and alleviate hypoxia during the development of CKD. Roxadustat, the first PHD inhibitor approved for clinical use in China, is used for the treatment of anemia in patients with CKD. Roxadustat treats anemia through multiple pathways, not only by activating HIF to increase EPO levels but also by decreasing hepcidin levels and reducing inflammation [146]. In addition, as PHD requires iron as a cofactor to hydroxylate the key prolines on HIF-α, desferoxamine and cobalt chloride as iron chelators can also activate HIF-α [47]. In an ischemic model of renal injury, cobalt oxide preconditioning can up-regulate the expression of EPO and vascular endothelial growth factor genes. Vascular endothelial growth factor is involved in vascular remodeling, and it protects the kidney from ischemia-reperfusion injury [147]. Cobalt therapy also blocked the hypoxia-induced renin-angiotensin system in this model [148]. However, the protective effect of HIF activation on the kidney is controversial. Studies have shown that blocking of HIF-1-induced miR-687 can have a protective effect on ischemic kidneys [149]. Similarly, metabolism switches to glycolysis and lipid accumulation from fatty acid utilization in DKD, SGLT2 inhibitors can correct metabolic disorders by reducing HIF-1α levels [150]. It has been suggested that HIF-2, not HIF-1, has protective effects on the kidney [151]. Interestingly, it has been shown that HIF-1 can protect mitochondrial dynamics by inducing miR-668 to inhibit mitochondrial protein 18 kDa [152]. On all accounts, these studies have demonstrated that HIF plays an important role in the pathogenesis of kidney disease, and the current controversy has focused on HIF-1; as for HIF-2, most studies are likely to view it as a protective factor. It is noteworthy that PHD inhibitors for current clinical use are all pan-PHD inhibitors and do not have specificity for a specific PHD isoform. Therefore, there is an urgent need to develop a drug that can selectively activate different subtypes of HIF, which may provide a safer and more effective therapeutic effect.

### 4.6. Fenofibrate

Fenofibrate is a common peroxisome proliferator-activated receptors (PPAR) agonist, belonging to the fibrate class of drugs, which is widely used to treat dyslipidemia. Lipid uptake, oxidation, and storage are modulated by the PPAR family of transcription factors, which control the expression of mitochondrial genes involved in lipid metabolism, such as NAD-dependent deacetylase sirtuin-3, medium-chain acyl-CoA dehydrogenase, and hydroxy methyl glutaril-CoA synthase-2 [153]. However, a growing number of clinical trials have shown that it has a protective effect on the kidney, reducing renal proteinuria and urea, and can be used in the therapy of DKD, hypertensive nephrosclerosis, and AKI [153–155]. Fenofibrate has been shown to promote fatty acid β oxidation, enhance mitochondrial metabolism and slow the growth of renal cysts in animal models of ADPKD [156]. Interestingly, statins that have lipid-lowering effects similar to the fibrate class of drugs did not show renal protection in the trial, so the benefits of fenofibrate may not be solely lipid-mediated but may be related to their antioxidant effect and mitochondrial protection [157]. In diabetic mice, fenofibrate increased the expression of adenosine monophosphate-activated protein kinase, which is involved in the regulation of glucose metabolism, lipid metabolism, ROS production, and intracellular eNOS levels [155]. Increasing the expression of eNOS can promote the generation of NO, which is an important regulator of vasodilation and mitochondrial respiration. Another experiment also demonstrated that PPAR activation inhibited angiotensin II induced nicotinamide adenine dinucleotide phosphate oxidase activation ROS production [154]. Furthermore, fenofibrate attenuated the changes in mitochondrial membrane potential and membrane structure induced by albumin, which played a part in maintaining the stability of mitochondrial function [153].

Reports of fenofibrate-associated increases in serum creatinine (SCr) levels have raised concerns regarding its deleterious effects on renal function. However, relevant studies have found that the increase in SCr caused by fenofibrate is transient and reversible even without the cessation of treatment. Their analysis concluded that fenofibrate-associated elevation of SCr arises from the changes in renal blood flow and glomerular pressure, rather than the renal function damage [158]. It is important to note that SCr level should be closely monitored in high-risk patients (older patients, patients with high dose and renal impairment). In summary, fenofibrate is beneficial for renal function.

### 4.7. Others

The therapy of ADPKD, beyond the fenofibrate, also can be administered with low doses of the glucose analog 2-deoxy-D-glucose (2DG). Treatment with 2DG restores normal renal levels of the phosphorylated adenosine monophosphate-activated protein kinase and its targeted acetyl-CoA carboxylase, inhibits glycolysis, and improves symptoms of ADPKD [159]. In addition, Long-term low-dose 2DG combined with metformin can also reduce eATP and lactic acid production and slow down the growth of renal cysts [160,161].

Pyruvate dehydrogenase, a key enzyme linking the glycolysis and tricarboxylic acid cycles, is particularly vulnerable to oxidative stress. In animal studies, acetyl-L-carnitine has been shown to inhibit renal pyruvate dehydrogenase decline in AKI and improve renal energy metabolism [162]. In other studies, L-carnitine has been found to promote free fatty acids into mitochondria for β-oxidation and prevent oxidative stress [163,164], and acetyl-L-carnitine can reduce oxidative damage to the kidney and protect renal function [165]. Currently, research and treatment of L-carnitine have focused on brain diseases, but it is also a potential drug to improve energy metabolism in the kidney.

## 5. Conclusion

Consistent clinical and research efforts have enabled further understanding of the role of abnormal energy metabolism in the development of chronic renal failure, and the therapies of energy metabolism are a worthy therapeutic target. Although at present, there is not any effective treatment for mitochondrial dysfunction caused by gene mutations, except for that caused by the lack of CoQ10. Mitochondrial replacement may be an effective replacement strategy, but there are ethical issues [166]. Furthermore, mitochondrial dysfunction caused by gene mutations is not only manifested in kidney function impairment, but also in the functional impairment of different systems such as muscles and nerves. Many potential agents are already under investigation for other indications in clinical trials. Disorders in renal energy metabolism can be further investigated in basic and clinical programs.

## Abbreviations

CKD: chronic kidney disease
GFR: glomerular filtration rate
ESKD: end-stage renal disease
DKD: diabetic kidney disease
OXPHOS: oxidative phosphorylation
DO2: oxygen delivery
QO2: oxygen consumption
RBF: renal blood flow
ROS: reactive oxygen species
mtDNA: mitochondrial DNA
nDNA: nuclear DNA
CoQ10: coenzyme Q10
PTCs: proximal tubule cells
TAL: thick ascending loop of Henle
eATP: extracellular ATP
ADP: adenosine diphosphate
CD73: ecto-5’-nucleotidase
PKD: polycystic kidney disease
P2X7R: P2X7 receptor
A_1_R: A_1_ receptor
A_2A_R: A_2A_ receptor
A_2B_R: A_2B_ receptor
A_3_R: A_3_ receptor
T1DM: type 1 diabetes mellitus
NO: nitric oxide
EPO: erythropoietin
RAS: renin-angiotensin system
AKI: acute kidney injury
HIF: hypoxia-inducible factor
Ang II: angiotensin II
eNOS: endothelial nitric oxide synthase
ADPKD: autosomal dominant polycystic kidney disease
SGLT2: sodium-glucose co-transporter 2
T2DM: type 2 diabetes mellitus
AT1: angiotensin II type 1
ARBs: angiotensin II type 1 receptor blockers
PHD: proline hydroxylase domain protein
FIH: factor inhibiting HIF
PPAR: peroxisome proliferator-activated receptors
Scr: serum creatinine
2DG: 2-deoxy-D-glucose
